# Antagonistic Mechanism of Metabolites Produced by *Lactobacillus casei* on Lysis of Enterohemorrhagic *Escherichia coli*

**DOI:** 10.3389/fmicb.2020.574422

**Published:** 2020-11-23

**Authors:** Arpita Aditya, Mengfei Peng, Alana Young, Debabrata Biswas

**Affiliations:** ^1^Department of Animal and Avian Sciences, University of Maryland, College Park, College Park, MD, United States; ^2^Biological Sciences Program, University of Maryland, College Park, College Park, MD, United States; ^3^Centre for Food Safety and Security Systems, University of Maryland, College Park, College Park, MD, United States

**Keywords:** antimicrobial effect, *Lactobacillus*, metabolites, membrane damage, EHEC, growth inhibition, antibacterial mechanism of action

## Abstract

Enhancing extracellular metabolic byproducts of probiotics is one of the promising strategies to improve overall host health as well as to control enteric infections caused by various foodborne pathogens. However, the underlying mechanism of action of those metabolites and their effective concentrations are yet to be established. In this study, we determined the antibacterial potential of the metabolites in the cell-free culture supernatant (CFCS) collected from wild-type *Lactobacillus casei* (LC_wt_) and genetically modified LC to overexpress linoleate isomerase (LC_CLA_). We also evaluated the mechanism of action of CFCSs collected from the culture of LC_wt_ in the presence or absence of 0.5% peanut flour (CFCS^wt^ and CFCS^wt+PF^, respectively) and LC_CLA_ alone (CFCS^CLA^) against enterohemorrhagic *Escherichia coli* (EHEC). The metabolites present in CFCS^wt+PF^ and CFCS^CLA^ eliminated EHEC within 24 and 48 h, respectively. Whereas CFCS^wt^ failed to eliminate EHEC but reduced their growth by 6.7 logs (*p* < 0.05) as compared to the control. Significant downregulation of the expression of cell division gene, *ftsZ*, supported the observed degree of bactericidal and bacteriostatic properties of the collected CFCSs. Upregulation of EHEC genes related to maintaining cell membrane integrity, DNA damage repair, and molecular chaperons indicated an intensive stress condition imposed by the total metabolites present in CFCSs on EHEC growth and cellular structures. A range of deviated morphological features provoked by the metabolites indicated a membrane-targeted action, in general, to compromise the membrane permeability of EHEC. The information obtained from this study may contribute to a more efficient prevention of EHEC related infections.

## Introduction

Most of the human and animal bacterial infections are treated following the anti-pathogen strategies targeting to either directly eliminate or control growth and proliferation of the pathogen in the host using conventional antibiotics and antimicrobial therapeutics (Callaway et al., 2013). However, the widespread application and/or misuse of antibiotics is raising the concern of antibiotic-resistant bacteria, which is limiting the effectiveness of the prophylactic application of the existing antibiotics (Llor and Bjerrum, 2014; Ventola, 2015). Besides, some bacterial infections, including enteric infections with enterohemorrhagic *Escherichia coli* O157: H7 (EHEC), cannot be treated with traditional synthetic antibiotics because of severe consequences of cytotoxic or adverse effects of the pathogen. For example, antibiotic therapy in EHEC infection can induce the rapid release of Shiga-like toxin (stx) in the gastrointestinal (GI) tract by the death of a vast number of pathogens, which may lead to hemolytic uremic syndrome (HUS) or kidney failure (Cordonnier et al., 2017; Aditya et al., 2019). Further, it also negatively modulates the normal inter-colonic microbial balance which increases the systemic absorption of the toxin (Peng et al., 2015a). Many other therapeutics, such as anti-motility agents, narcotic analgesics, and anti-inflammatory non-steroidal drugs which may alleviate diarrhea, pain, or fever, is also suggested to be avoided in treating EHEC infections because of their observed association with HUS development and lowering the renal blood flow (Cimolai et al., 1992; Murray and Brater, 1993; Bell et al., 1997).

Recent research focusing on the pro-commensal strategy to control foodborne bacterial infections is gaining attention (Callaway et al., 2013). The pro-commensal approach aims to promote the growth of probiotics (e.g., *Lactobacillus* spp., *Bifidobacterium* spp., *Streptococcus* spp., etc.) and suppress the pathogen number or growth in the gut microbial ecosystem (Fuller, 1989; Ouwehand et al., 2002). Adding a specific nutritional component for beneficial microbes, i.e., prebiotic or prebiotic-like dietary products (colloquially termed as “health-foods” originating from the plant or animal sources, e.g., berry fruits, cocoa, peanut, green tea, etc.), confers selective growth benefits to probiotics and result in the production of a diverse array of their metabolic byproducts (Fuller, 1989; Schrezenmeir and de Vrese, 2001; Reid and McCormick, 2002; Charernjiratragul et al., 2010). In the gut environment, probiotics exert their beneficial potential by competitively excluding the pathogens/opportunistic pathogens, preventing pathogenic adhesion to mucosal surfaces and colonization. The most promising feature of probiotics already established from *in vitro* and *in vivo* studies is their antimicrobial activity which is conferred through their metabolic byproducts (Saarela et al., 2000; Mercenier et al., 2008; Kechagia et al., 2013; Ming et al., 2018; Peng et al., 2018). Probiotics alone or in combination with prebiotics (known as a synbiotic strategy) produce many cell-associated and extracellular molecules as well as bioactive metabolites through their normal physiological processes. The formation of these compounds depends on the substrate, population density, particular species and strains of probiotics, and their kinetics (Delgado et al., 2007; Jones et al., 2008). The antibacterial potential of probiotics has been proven to be a concerted effect of these compounds against pathogen growth. Detrimental effects against the growth of both Gram-positive and Gram-negative foodborne pathogens, such as *Listeria monocytogenes*, *Campylobacter jejuni*, *Enterococcus faecalis*, *Salmonella enterica*, EHEC, *Vibrio parahaemolyticus*, and *V. cholerae* are observed to be potentially controlled by pro-commensal strategy (Jones et al., 2008; Mezaini et al., 2009; Charernjiratragul et al., 2010; Nigatu et al., 2015; Ren et al., 2018).

Probiotics have been reported to produce various metabolites, such as flavonoids, glycosyl compounds, steroids, indole, indazole, benzoic acid, gluco-phospholipid, catechol, hydrocinnamic acid, salicylic acid, ferulic acid, caffeic acid, lactic acid, acetic acid, formic acid, linoleic acid, phenyllactic acid, vanillic acid, azelaic acid, hydrocoumaric acid, hydroferulic acid, hydrocaffeic acid, 2,3-butadione, reuterin, acetaldehyde, hydrogen peroxide (H_2_O_2_), hydroxyl radical, peptides, or proteins, such as the bacteriocins, and countless derivatives of these compounds (Cleveland et al., 2001; Schnürer and Magnusson, 2005; Broberg et al., 2007; Peng et al., 2015a). Besides foodborne illnesses, many of the lifestyle-based human health complications, e.g., cardiovascular disease, diabetes, obesity, etc. can be prevented and more efficiently treated with a combination of existing drug therapy and healthy dietary choice (Drexler and Institute of Medicine (US), 2010; Ramalingum and Mahomoodally, 2014). Consumption of probiotic bacteria in fermented dairy products or as a supplement has become very popular due to their reported valuable association with lactose intolerance, improved gut health which is linked to the decreased risk of various other complications of the GI tract like irritable bowel disease (IBD), celiac disease, etc. (Salminen et al., 2005; Kechagia et al., 2013; Ramalingum and Mahomoodally, 2014). Similarly, many of the probiotics originated bioactive metabolites, e.g., linoleic acid isomers or conjugated linoleic acid (CLA), have been reported to possess potential anti-carcinogenic, anti-inflammatory, and anti-microbial activities (Banni, 2002; Belury, 2002; Peng and Biswas, 2017). Apart from dietary sources, this essential omega-6 fatty acid is obtained from microbial biosynthesis of many probiotic species, e.g., *Bifidobacterium* spp., *Lactobacillus acidophilus*, *L. brevis*, *L. casei*, *Corynebacterium* spp., etc. (Ogawa et al., 2001; Kishino et al., 2002).

Furthermore, the quantity and quality of metabolites generated by *L. casei* could be improved by peanut flour, and they exhibit more intensive antimicrobial effects towards the growth of common foodborne pathogens (Peng et al., 2015a). However, the mechanism by which these metabolites work against pathogens and their effective concentrations are yet to be elucidated. In this study, we aimed to investigate the underlying mechanism of extracellular metabolites, including CLA, collected from the culture of *L. casei* strains in various conditions against EHEC growth, their cell membrane integrity as well as genomic DNA.

## Materials and Methods

### Bacterial Strains and Their Growth Conditions

In this study, Shiga toxin-producing enterohemorrhagic *E. coli* O157: H7 EDL933 (EHEC EDL933) (ATCC700927) was used as a representative of a foodborne pathogen. This bacterium was grown at 37°C overnight on Luria-Bertani (LB) agar or in LB broth (Becton, Dickinson and Co., Sparks, MD, United States) as required under aerobic conditions (Thermo Fisher Scientific Inc., Waltham, MA, United States). Two probiotic strains, including wild type *Lactobacillus casei* (LC_wt_) (ATCC334) and an engineered linoleate isomerase over-producing *L. casei* (LC_CLA_), previously generated in our laboratory (Peng et al., 2018) were grown at 37°C in aerobic conditions on de Man-Rogosa-Sharpe (MRS) agar or in MRS broth (Merck KGaA, Darmstadt, Germany) in a CO_2_ incubator (Thermo Fisher Scientific Inc., Waltham, MA, United States) as required.

### Peanut Flour Preparation and Modulating Metabolites From Probiotics

Jumbo Virginia in-shell peanuts (*Arachis hypogaea*) packed and labeled by Royal Oak Peanuts (Drewryville, VA, United States) was purchased from a local vendor, unshelled, and the red skin was removed by hand to collect the raw white kernel parts. Later, they were manually grounded into a fine powder and a 10% (w/v) peanut suspension in sterile deionized water was prepared by overnight stirring. Following aseptic techniques, the suspension was sieved by a kitchen fine mesh sieve strainer (LiveFresh, Darwen, United Kingdom) to separate larger peanut fractions from the peanut suspension. The pH of the sieved peanut suspension was measured and sterilized under UV irradiation for 3 h and cultured it on LB agar to ensure sterility. Later, this suspension was added in MRS broth to achieve a final concentration of 0.5% (v/v) to naturally modulate the metabolites of LC_wt_.

### Culture Condition of *Lactobacillus* Strains and Collection of Cell-Free Culture Supernatants (CFCSs)

Cell-free culture supernatants (CFCSs) were collected from the individual cultures of *Lactobacillus* strains in MRS broth as described previously by Peng et al. (2015b) with slight modification. Briefly, 0.5% (v/v) whole peanut flour suspension was added as supplementation in MRS broth to stimulate the growth of LC_wt_ and enhance its metabolite production. Approximately 10^6^ colony-forming units (CFU)/mL of LC_wt_ were inoculated to the fresh MRS broth with or without peanut flour and the metabolites in CFCSs were collected at 48 h of incubation by centrifuging at 4,000 × *g* for 20 min, followed by filtration of the supernatant with a sterile syringe filter (0.2 μm pore size) (VWR International, Radnor, PA, United States). LC_CLA_ was grown without peanut flour and CFCS was collected following the same procedure. In all conditions, we cultured the *L. casei* strains (LC_wt_, LC_wt + PF_, and LC_CLA_) under aerobic condition in 5% CO_2_ incubator. The collected CFCSs from LC_wt_, LC_wt_ in presence of 0.5% peanut, and LC_CLA_ were labeled as CFCS^wt^, CFCS^wt+PF^, and CFCS^CLA^, respectively, and preserved at 4°C until further use.

### Assay of EHEC EDL933 Inhibition With CFCSs Collected From LC_wt_ or LC_CLA_

The effect of the collected metabolites against EHEC EDL933 growth was determined quantitatively *in vitro* following the method previously described in triplicate (Peng et al., 2015a; Salaheen et al., 2016; Aditya et al., 2019). Briefly, individual culture tubes containing LB broth and one of the CFCSs (CFCS^wt^, CFCS^wt+PF^, or CFCS^CLA^) in a ratio of 3:1 (v/v) was used as a treatment, where the same ratio of LB and MRS broth was considered as control. For the initial pathogen load, the optical density of EHEC EDL933 was fixed at 0.1 at 600 nm (OD_600_) by spectrophotometer (PerkinElmer, Waltham, MA, United States) which was further diluted by 100-fold to achieve a suspension of ∼10^4^ CFU/mL. A total of 50 μL of the diluted EHEC EDL933 suspension was inoculated to control and treatment tubes, each having a final volume of 5 mL. The number of viable cells in the presence of each type of CFCS was estimated at 4, 8, 12, 24, and 48 h time points by serial dilution in phosphate-buffered saline (PBS; pH 7.4), followed by plating on LB agar.

### Quantitative Assay for Gene Expression of EHEC EDL933 Treated With CFCSs

For comparing EHEC EDL933 gene expressions in the presence or absence of CFCSs, RNA extraction, and cDNA synthesis were executed in triplicate as previously described (Salaheen et al., 2016; Peng et al., 2018). Using the cDNA (40 ng) as a template, the q-PCR reaction mixture was prepared according to PerfeCTa^®^ SYBR^®^ Green FastMix^®^ protocol (Quanta Biosciences, Beverly, MA, United States) and amplified in an Eco Real-Time PCR system (Illumina, San Diego, CA, United States) with 30 s denaturation at 95°C, followed by 40 cycles of 95°C for 5 s, 55°C for 15 s, and 72°C for 10 s. The relative expression level of the target genes in treatment was calculated by the comparative log fold change. The *C*_t_ (cycle threshold) value of target genes in treatment was normalized to the reference gene (Aditya et al., 2019) (all genes used in this study are listed in Table 1) in treatment then the relative expression of the genes was compared between respective treated and untreated conditions (Livak and Schmittgen, 2001).

**TABLE 1:** Primers used for RT-qPCR analysis.

Function	Gene	Primer sequence (5′–3′)	References
Housekeeping gene	16 S rRNA	F: CGTTACCCGCAGAAGAAGC	Aditya et al. (2019)
		R: GTGGACTACCAGGGTATCTAATCC	
Cell division protein	*FtsZ*	F: TTGGGTATCCTGACCGTTGC	Used in this study
		R: AGCAGTTTGTCGTTCGGGAT	
Membrane heat shock proteins	*HtpX*	F: GGCAACCCGCTGATCTACTT	Used in this study
		R: AGCGCGGCAATCATTTTCTC	
	*cpxP*	F: TTTCTGCGGTGACAAGACGA	Used in this study
		R: TCAGGCGATAACTGGCATCC	
	*pspA*	F: ATCCACAGAAACTGGTGCGT	Used in this study
		R: GTTTCTTTTCTGCCAGCGCA	
	*pspB*	F: ACCGATCTGGTTATGGCTGC	Used in this study
		R: TTCCAGCGCCTGAATACGTT	
	*pspC*	F: GTGCGTATCCTGGTGGTGTT	Used in this study
		R: CACCAAAGGCCATGTTGTCC	
	*pspE*	F: AGTGAAAGAGCGCATTGCCA	Used in this study
		R: CGTGGGTATATCCCATCTCGC	
SOS response and DNA replication related genes	*recA*	F: CAGGCAGTTGCATTCGCTTT	Used in this study
		R: TCTACGGCGAACTGGTTGAC	
	*lexA*	F: GGTCGTTGTCGCACGTATTG	Used in this study
		R: CTGCTGACGAAGGTCAACGA	
	*tus*	F: TCCTGGCACAGCTGGAAAAA	Used in this study
		R: ACTTCGCGTTCTGTGGTAGG	
	*yebG*	F: CATAGCGAAAGGGCTTCACG	Used in this study
		R: AGCAAAAAGGAAGCCGATGC	
Chaperon proteins	*ibpA*	F: CGACGAACAAAAAGAGCGCA	Used in this study
		R: ACCAGGTTAGCACCACGAAC	
	*ibpB*	F: AGCGACGATAACCACTACCG	Used in this study
		R: GCCCTTGATGCAGCCATTTT	
	*clpB*	F: GGCCGAGGAACAGGAATGAA	Used in this study
		R: ATTGGTCAGAACGAAGCGGT	
	*grpE*	F: GCACAACATCCAGCATCGAC	Used in this study
		R: TGCCGGTGATTGATAGCCTG	
	*dnaJ*	F: TCATGGTTCTGGTCAGGTGC	Used in this study
		R: TGCCGGGATTTTAACGGACA	
	*dnaK*	F: GAAAGTTGCACTGCAGGACG	Used in this study
		R: GGTTAACGTCTTTACGCGGC	

### Fluorescence Microscopy of EHEC EDL933 Treated With CFCSs

The live/dead cells of EHEC EDL933 treated with CFCSs was determined using the BacLight^TM^ bacterial viability kit (L7012) (Molecular Probes, Inc., Eugene, OR, United States) following the standard protocol provided by the vendor (Farkas et al., 2017). Briefly, the optical density of the bacterium was fixed at (OD_600_) 0.2 in LB broth which was treated with 25% (v/v) of CFCS^wt^, CFCS^wt+PF^, and CFCS^CLA^ separately. For control, bacterial cells were grown in LB broth mixed with 25% (v/v) MRS broth. The control and treatments were cultured at 37°C under aerobic conditions. At 4, 24, and 48 h time points, 1 mL of bacterial suspension from each culture tube was collected, washed three times, and resuspended in PBS. Then the cells were stained with an equal volume of SYTO 9 and propidium iodide (PI) dye. After incubating in the dark for 15 min, 5 μL of the stained bacterial suspension was taken on a microscope slide and fixed with 0.5% (w/v) agarose. A coverslip was swiftly placed on the specimen and visualized under Zeiss AxioObserver fluorescence microscope (Zeiss, White Plains, NY, United States) with 100× oil immersion objective lens. GFP and DsRed filter sets were used to capture images of the specimens.

The remaining stained bacterial suspension was analyzed for the fluorescence intensity by a Cytation 5 spectrophotometer (BioTek Instruments, Inc. Winooski, VT, United States) at 530 and 630 nm for SYTO 9 and PI, respectively. For this step, EHEC EDL933 treated with isopropanol was used as a positive control to confirm cell death.

### Scanning Electron Microscopy (SEM) of EHEC EDL933 Cells Treated With CFCSs

The optical density of EHEC EDL933 culture was measured and adjusted at 0.2 (OD_600_) in LB broth and incubated overnight at 37°C under aerobic conditions in the presence of either 25% (v/v) of MRS broth (served as a control), CFCS^wt^, CFCS^wt+PF^, or CFCS^CLA^. Both control and CFCSs treated EHEC EDL933 cells were harvested and washed with PBS three times. After that, cells were fixed with 2.5% (v/v) glutaraldehyde (Electron Microscopy Sciences, Hatfield, PA, United States) for 1 h. Five microliters of the fixed bacterial suspension were taken and spread on a polycarbonate membrane filter (pore size 0.2 μm) (Millipore Sigma, Burlington, MA, United States). Then the membranes were washed thrice with PBS followed by dehydration with a series of increasing aqueous ethanol concentrations, each for 15 min [30, 50, 70, 80, and 100% (v/v)] (Farkas et al., 2017). The membranes were stored overnight under anhydrous calcium sulfate (Peng et al., 2018). The EHEC EDL933 cells on the membrane were gold coated and observed under Hitachi SU-70 FEG scanning electron microscope (Hitachi Ltd., Japan) at an accelerating voltage of 10 kV.

### DNA Degradation Assay

From an overnight culture of EHEC EDL933 (inoculum size ∼10^4^ CFU/mL) in LB broth, 1 mL of the bacterial suspension was harvested and quickly spinned to collect the pellet. Genomic DNA was extracted from this pellet with Trizol^TM^ reagent (Invitrogen, Waltham, MA, United States) according to the Trizol Reagent (DNA isolation) user guide (Chomczynski and Sacchi, 1987). The extracted genomic DNA was treated with CFCSs to investigate their DNA damaging potential (Brudzynski et al., 2012). DNA (90 μg/mL) was mixed with CFCSs in a ratio of 3:1 and incubated at 37°C for 4 h. DNA treated with LB/MRS broth in the same ratio considered for the control. After the treatment, 1 μL of the 5× loading dye was added, and the mixture was loaded onto a 1% agarose gel. Gel electrophoresis was performed according to a protocol described previously (Brudzynski et al., 2012).

### Statistical Analysis

SAS 9.2 software (SAS Institution Inc., Cary, NC, United States) was used to determine the statistical significance. The one-way analysis of variance (ANOVA) followed by Dunnet test was applied to determine significant differences of EHEC EDL933 growth and gene expression levels between control and treatment based on a significance level of 0.05.

## Results

### Growth Inhibitory Effect of CFCSs on EHEC EDL933

**FIGURE 1:**
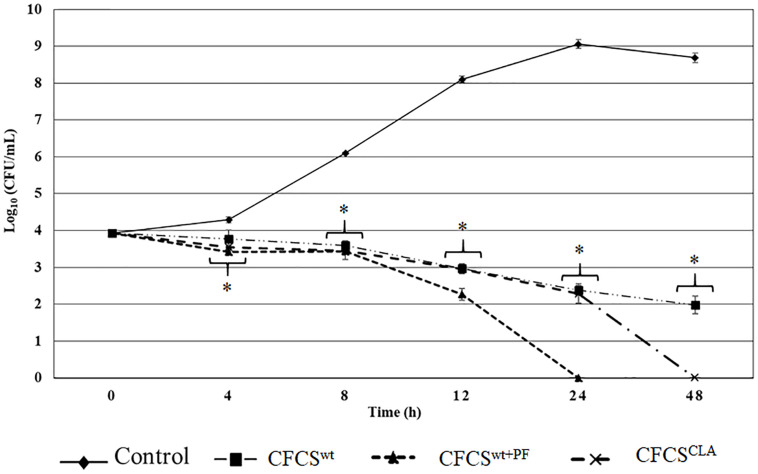
Antagnostic effect of crude metabolites (CFCS^wt^, CFCS^wt+PF^, and CFCS^CLA^) against the growth of EHEC EDL933 over 48 h time. The bars represent the average ± standard deviation among replicates and the asterisks (^∗^) indicate a significant difference between control and treatment (*p* < 0.05).

Quantitative assessment of the antagonistic effect of the metabolites collected in CFCS^wt^, CFCS^wt+PF^, and CFCS^CLA^ exhibited statistically significant bactericidal property against EHEC EDL933 as compared to the control (Figure 1). Metabolites of LC_wt_ which was enhanced by peanut flour supplement (CFCS^wt+PF^) exhibited the strongest inhibitory potential by eliminating EHEC EDL933 growth within 24 h, whereas metabolites collected from LC_wt_ without the supplement (CFCS^wt^) could not eradicate the pathogen even after 48 h of the treatment. However, a continuous bacteriostatic effect was observed on CFCS^wt^, which reduced the growth of pathogen by 6.7 logs (*p* < 0.05) as compared to the control at 48 h. The CFCS collected from LC_CLA_ (CFCS^CLA^) which contained more CLA than the other two CFCSs (CFCS^wt^ and CFCS^wt+PF^) exhibited a stronger bactericidal effect than CFCS^wt^ against EHEC EDL933 by eradicating its growth within 48 h (Figure 1).

The antagonistic effect of the CFCSs on the growth of EHEC EDL933 was also observed at both lower and higher concentrations. A lower concentration of all the collected CFCSs (e.g., 10% v/v) exerted a bacteriostatic effect on the pathogen which was attenuated after 4–8 h depending on the *Lactobacillus* strains and the growth condition specifically the presence or absence of peanut flour as a growth supplement. Whereas a higher concentration (e.g., ≥50% v/v) of CFCSs regardless of the cultural conditions eradicated EHEC EDL933 within 12 h as compared to the negative control (without CFCS, data not shown).

### Alteration of EHEC EDL933 Gene Expression Indicates Adverse Growth Environment

**FIGURE 2:**
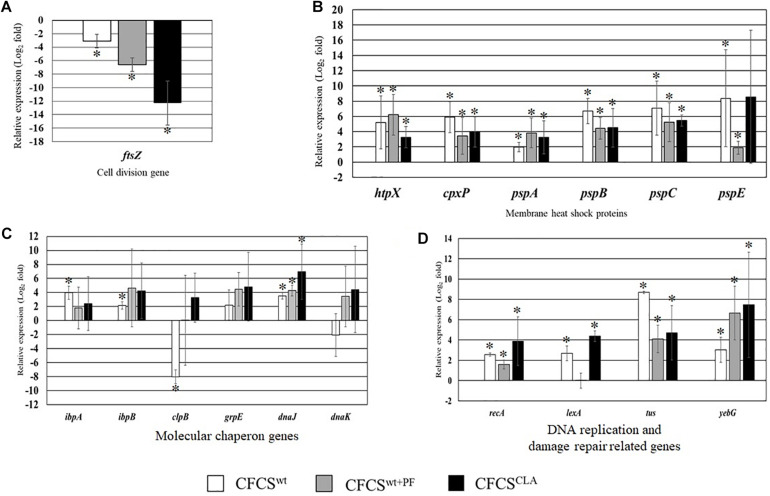
Relative expression of genes related to EHEC EDL933 cell division **(A)**, membrane heat shock proteins **(B)**, various molecular chaperon genes **(C)**, and DNA replication and damage repair-related genes **(D)**. The bars represent relative fold-change between control and individual treatment. The error bars are showing the ± standard deviation among replicates, and the asterisks (^∗^) indicate a significant difference between control and treatment (*p* < 0.05).

The relative expression of several critical genes of EHEC EDL933 cells, specifically the genes involved in cellular structure and cell-division, were examined in the presence of CFCS^wt^, CFCS^wt+PF^, and CFCS^CLA^ (Figure 2). The most crucial gene for bacterial cell division, *ftsZ* which binds with the Z-ring to facilitate cell division was significantly downregulated (Figure 2A) in the presence of all of three CFCSs but the suppressive effect by CFCS^CLA^ was more intense than the other two CFCSs (CFCS^wt^ and CFCS^wt+PF^), indicating the presence of metabolites produced by LC_CLA_ is important but their concentration, possibly CLA, is critical in intensive inhibition of growth and cell division of EHEC EDL933.

On the other hand, genes associated to maintain membrane structure (*htpX*, *cpxP*, *pspA*, *pspB*, *pspC*, and *pspE*) of EHEC EDL933 were upregulated due to the treatment with CFCSs collected from LC cultures (Figure 2B). We found that expression of these genes was upregulated at various folds ranging from 2 to 8.2 which could be linked to the notion of membrane targeting activity of the metabolites. We also evaluated the expression of some genes known as molecular chaperones (*ibpA*, *ibpB*, *clpB*, *grpE*, *dnaJ*, and *dnaK*) which are expressed in stress as well as in non-stressed conditions. In this study, CFCS^wt+PF^ and CFCS^CLA^ upregulated the expression of all chaperon genes that were tested, however in the presence of CFCS^wt^, *clpB* and *dnaK* genes were found to be downregulated, indicating that the effect is attributed to the variation in CFCS composition in terms of concentration (Figure 2C). The upregulation of the DNA replication and SOS response-related genes (*recA*, *lexA*, *tus*, and *yebG*) indicated that the CFCSs regardless of the source might also have a DNA damaging activity (Figure 2D) arising from the metabolites.

### Membrane Integrity of EHEC EDL933 Is Compromised by CFCSs

**FIGURE 3:**
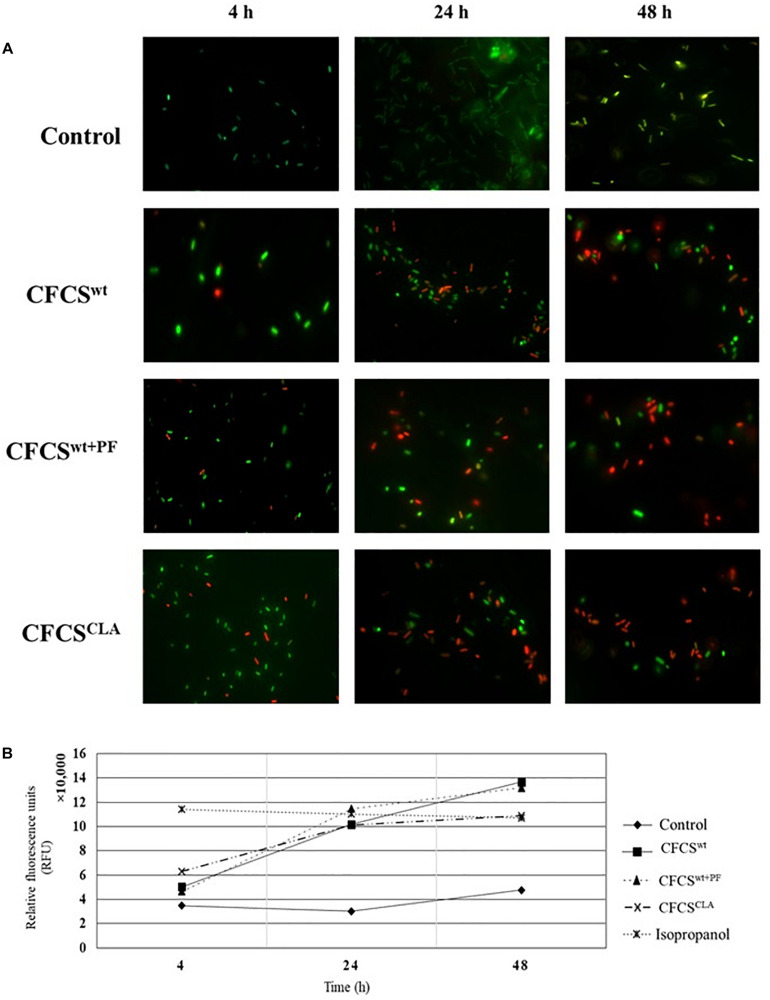
Fluorescent micrographs showing altered EHEC EDL933 cell membrane permeability **(A)** in the presence of *Lactobacillus* metabolites. An increase in the extent of membrane disintegration was visible because of the presence of CFCS^wt^, CFCS^wt+PF^, and CFCS^CLA^ throughout 4, 24, and 48 h. The green fluorescence represents bacterial (EHEC EDL933) cells with an intact membrane whereas red fluorescence indicates a disrupted membrane. Increasing the intensity of red fluorescence (relative fluorescence units, RFU) over the incubation period for treatments indicated an increasing proportion of dead cells in the cultural conditions **(B)**.

In membrane integrity assay, we observed that the extracellular metabolic byproducts of *L. casei* strain present in CFCS^wt^, CFCS^wt+PF^, and CFCS^CLA^ influenced the normal cell membrane permeability of EHEC EDL933 cells by directly disrupting the membrane integrity (Figure 3). To compare the membrane damage, the CFCS treated and non-treated EHEC EDL933 cells were visualized after staining with fluorescent nucleic acid dyes SYTO 9 and PI. In general, green fluorescence is observed from a population when stained with SYTO 9, since the dye stains the DNA of both bacteria with the intact and damaged membrane. On the other hand, PI can penetrate bacterial cells only when the normal cell membrane integrity is compromised. When co-stained the red fluorescence overrides the green one because of their stronger affinity to DNA than SYTO 9. We observed a significant progression in cell membrane disruption by all treatments over the 48 h period (Figures 3A,B), which was reflected by the progressive abundance of red fluorescence as compared to the control. Fluorescent micrographs (Figure 3A) of the CFCSs-treated cells showed an increasing number of red fluorescent cells with the progression of treatment time (up to 48 h). A discernable variation in the number of red fluorescent cells was observed among the treatments. A similar proportion of damaged cells were observed from CFCS^wt+PF^ and CFCS^CLA^ at 48 h while CFCS^wt^ had comparatively less damaged cells at the same time point. However, a significant number of green fluorescent EHEC EDL933 cells were detected under the microscope while the growth inhibition assay showed complete elimination of the pathogen. These findings can be related to the diverse mechanism of growth-inhibitory action of CFCSs besides bacterial cell membrane disruption that arises due to the diversity and concentration of metabolites present in CFCSs.

The spectrophotometric observation (Figure 3B) of the CFCS treated EHEC EDL933 cells showed a growing proportion of membrane damaged cells which were reflected by the increasing red fluorescence (at 630 nm wavelength). For this assay, EHEC EDL933 cells treated with 70% isopropanol was used as a positive control for bacterial membrane damage as isopropanol lyse bacterial cells by dissolving the membrane. The fluorescence from the isopropanol treated EHEC EDL933 cells remained static throughout the experiment because of their rapid mode of action. On the other hand, red fluorescence of EHEC EDL933 cells cultured without CFCSs was significantly lower than other treatments that exactly coincided with the fluorescent microscopy and growth inhibition experiment.

### Morphological Deviation of EHEC EDL933 Membrane Treated With CFCSs

**FIGURE 4:**
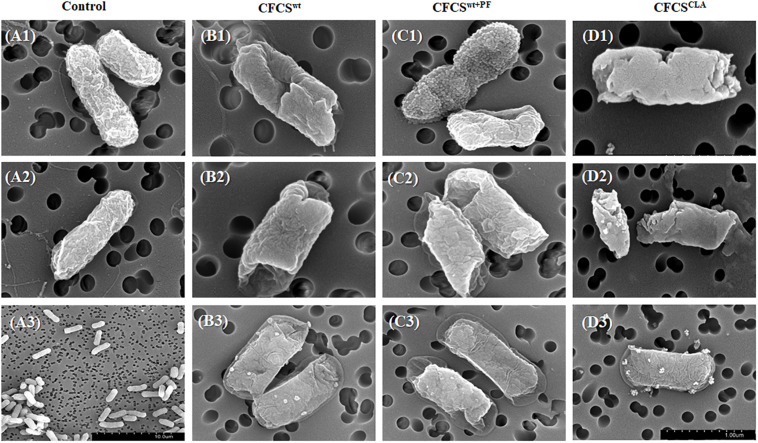
Scanning electron micrographs showing the effect of metabolites in CFCSs on the EHEC EDL933 cell surface after overnight (18 h) incubation. No membrane disruption was visible on the control group **(A1–A3)**, whereas dents were apparent at the ends of the cells **(B1, B2, C2)**. Apart from the dents, bleb-like structures were also visible **(C1)**, while a crumpled and blistery surface was also observed **(D1–D3)**. In some cells, the plasma membrane was displaced from the cell wall **(B3, C3)**.

The effects of the CFCSs collected from different cultural conditions, i.e., CFCS^wt^, CFCS^wt+PF^, and CFCS^CLA^ on EHEC EDL933 membrane showed significant divergence (Figure 4). The morphology of CFCS-treated EHEC EDL933 cells was visualized to compare the effect of metabolites and their concentration using SEM. The electron micrographs of overnight (18 h) CFCSs-treated cells showed distinct morphological attributes of the membrane as compared to the control. Untreated EHEC EDL933 cells’ morphology was undamaged (Figures 4A1–A3), while all the CFCS-treated EHEC EDL933 cells exhibited a diverse array of membrane disruption and deviation from the normal structure. The metabolites present in CFCS^wt^ disrupted both outer and inner membranes (IMs) from random directions which made a hollow opening on the pathogen (Figures 4B1,B2). In addition to the membrane disruption, a bleb-like structure was observed uniquely when the pathogen was treated with CFCS^wt+PF^ (Figure 4C1). Whereas, some EHEC EDL933 cells exhibited a crinkly appearance with a smooth surface in the presence of CFCS^CLA^ (Figures 4D1,D2). This particular appearance was also uniquely seen in CFCS^CLA^ treated cells which might be the concerted effect of CLA along with other metabolites. Dislocation of the plasma membrane from the bacterial cell wall was observed in all three types of CFCS treated EHEC EDL933 cells (Figures 4B3,C3,D3). Although no obvious disruption of the outer membrane was apparent in these cells, the cytoplasmic content was seemed to be released possibly through a porous opening on the displaced membranes.

### DNA Degradation by CFCSs

**FIGURE 5:**
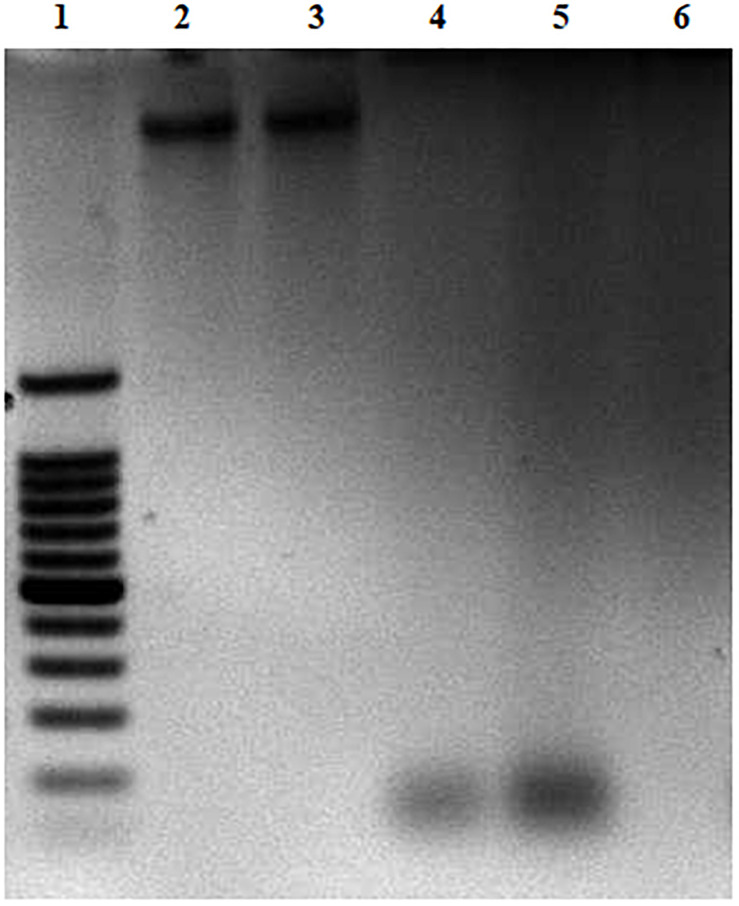
DNA degradation of EHEC EDL933 treated in the presence of CFCSs for 4 h. For the agarose gel: Lane 1 represents a 100 bp DNA ladder. Lane 2 represents EHEC EDL933 genomic DNA without any treatment. Lanes 3–6 represent EHEC EDL933 genomic DNA separately treated with 25% (v/v) of LB/MRS broth mixture (control), CFCS^wt^, CFCS^CLA^, and CFCS^wt+PF^, respectively.

The effect of CFCSs on EHEC EDL933 genomic DNA was investigated by agarose gel electrophoresis. All the collected CFCSs (CFCS^wt^, CFCS^wt+PF^, and CFCS^CLA^) were found to be destructive to the DNA of EHEC EDL933 within the 4 h of treatment (Figure 5). However, an interesting fact was observed from CFCS^wt+PF^ since there was no visible band in the lane (Figure 5, lane 6) while CFCS^wt^ and CFCS^CLA^ lanes showed bands at around 100 bp. This might be connected to the presence of metabolites that are coming from the peanut flour.

This study was also performed with pH adjusted CFCSs. The original pH of the CFCS^wt^, CFCS^wt+PF^, and CFCS^CLA^ were 3.71, 3.48 and 3.75, respectively. After adjusting the pH to 5.43 [the pH of 3:1 (v/v) mixture of LB and MRS broth], the same effect was recorded as of pH non-adjusted CFCSs (data not shown). Therefore, this observation indicated that the destruction of DNA was not the effect of acidic pH rather the concentration of metabolites present in the CFCSs.

## Discussion

The growth of probiotics and their production ability of extracellular metabolites can be enhanced by supplementing prebiotics which is indigestible by the hosts, such as inulin, fructooligosaccharides, pectic oligosaccharides, oligofructose, and polyphenols in both culture condition and feed/food (Rurangwa et al., 2009; Pranckutė et al., 2016). A similar effect has been observed by supplementing prebiotic-like foods known as functional foods, such as peanut, cocoa, or berry fruits (Peng et al., 2015a; Salaheen et al., 2016; Tabashsum et al., 2018). The nourishing effects of functional foods on human health are associated with the content and concentration of fatty acids, plant-based protein, vitamins, minerals, fiber, antioxidants, sterols, flavonoids, and phenolics (Grosso et al., 2015; Arya et al., 2016; Lamuel-Raventos and Onge, 2017). However, sometimes some of these components may be linked to undesirable conditions, e.g., α-arachin protein present in peanut white kernel is thought to be the cause of peanut allergy (Johns and Jones, 1916; Barnett et al., 1983). Besides, fungal infestation, e.g., *Aspergillus flavus* on grainy functional foods is linked to aflatoxin mediated carcinogenesis (Achar et al., 2009). Considering these adverse effects, cost, availability, and persistent applicability all prebiotics cannot always stimulate the growth and metabolite production of probiotics for hosts. Hence, engineered probiotics capable of producing critical metabolites without the presence of prebiotic components are an alternative option (Peng et al., 2018). Further, such type of modified probiotic strains could be more effective in the fermentation of dairy products and vegetables. In this study, we qualitatively compared the specific effects arising from the metabolites of a probiotic bacterium when it grows alone, in presence of a prebiotic-like supplement, and aimed to achieve a similar beneficial effect by genetically engineering a probiotic strain through overproducing one of the bioactive metabolites, CLA.

In agreement with Peng et al. (2018) reported previously, we also observed a gradual decline in the growth of EHEC exerted by all collected CFCSs from *L. casei* strains in a time-dependent manner (Peng et al., 2015a, 2018). However, CFCS^wt+PF^, collected from the *L. casei* in the presence of peanut flour, exhibited a stronger inhibitory effect than CFCS^wt^ as well as CFCS^CLA^, as expected. By genetic modification of *L. casei*, we could increase the conversion of CLA only 21 folds (Peng et al., 2018) while in the presence of peanut flour (0.5%) they might have produced more CLA and other metabolites as well. Hence, CFCS^CLA^ which contained a higher amount of CLA inhibited the growth of EHEC more efficiently than CFCS^wt^ which was collected from wild-type *L. casei* without the peanut flour supplement. Lamuel-Raventos and Onge (2017) reported that nutritionally indigestible components of peanuts like polymerized polysaccharides and polyphenols are enzymatically converted by gut microbes into a diverse array of metabolic byproducts encompassing organic acids, fatty acids, and H_2_O_2_, etc. (Lamuel-Raventos and Onge, 2017) that might have conferred the relatively rapid antagonistic effect of CFCS^wt+PF^ against EHEC growth in our study.

Previously, we also reported that CFCS^CLA^ impaired physicochemical properties of bacterial pathogens such as auto-aggregation, surface hydrophobicity, and biofilm formation of several enteric bacterial pathogens more efficiently than CFCS^wt^ (Vu et al., 2009; Peng et al., 2018; Tabashsum et al., 2018). As the physicochemical properties play a major role in pathogenic bacterial adherence to host cells, secretion of effector proteins, and initiate colonization (Sorroche et al., 2012; Cordonnier et al., 2017; Aditya et al., 2019), we also compared the expression of genes which are involved in such physicochemical properties and secretion of proteins in the presence of CFCSs collected from LC_wt_ and LC_CLA_. Major virulence genes involved in EHEC Type-III secretion system (T3SS), i.e., *eaeA*, *espA espB*, *espD*, *tir*, and *ler* are downregulated presumably by the polyunsaturated fatty acid components of CFCSs (Cardenal-Muñoz and Ramos-Morales, 2011; Peng and Biswas, 2017; Peng et al., 2018). Altered physicochemical properties and virulence gene expression of EHEC are concurred with its reduced adherence to host intestinal epithelial cells. A paucity of receptor-like molecules on the host cell surface is one of the reasons for reduced adherence since the receptors are already settled with the metabolites present in CFCSs (Bernet et al., 1994; Matsuo et al., 2012; Peng et al., 2015b).

It is already explicitly established that the components of CFCSs create an adverse condition that induces the pathogen to adaptively regulate its genes in response to such stressful conditions for their survival (Wall et al., 2004; Seshasayee et al., 2009). We observed a significant downregulation of *ftsZ*, the key regulator of EHEC cell division, which coincides with our observed bacteriostatic effect of CFCSs regardless of the source in the first 5 h of treatment. In an unfavorable growth environment, such as nutrients insufficiency, adverse pH, and presence of growth inhibitory compounds bacteria usually delay their proliferation due to the inhibition of septal ring (Z-ring) proteins specifically FtsZ or DnaA or disruption of other factors involved in Z-ring formation (Arends and Weiss, 2004; Jonas, 2014). When the Z-ring is unable to form septa, the cells continue to grow like long aseptate filaments which may not form colonies upon plating (Addinall et al., 1997; Vicente et al., 2006). In this study, a stronger suppression on *ftsZ* gene was observed when the EHEC EDL933 cells were treated with CFCS^CLA^ which might be correlated to the higher concentration of metabolites including CLA.

In a stressful environment, bacterial cells produce defective and misfolded membrane proteins much higher than they would produce in a favorable growth condition. To retain membrane integrity, the bacterial cells need to correct or destroy the misfolded proteins (Sakoh et al., 2005). In our study, we observed an overexpression of *htpX* and *cpxP* genes which are involved in the quality control of membrane proteins of EHEC in the presence of CFCSs. We also observed an upregulation of genes that are involved in phage shock protein operon (*pspA*, *pspB*, *pspC*, and *pspE*). Yoshitani et al. (2019) found that *htpX* along with *ftsH* lyse the abnormal IM proteins of EHEC (Yoshitani et al., 2019). Besides, Shimohata et al. (2002) reported that the accumulation of misfolded proteins in the IM may occur due to the inefficiency or loss of *htpX* or *ftsH* which further activates Cpx stress response proteins located in periplasmic space (Shimohata et al., 2002). The Psp proteins are thought to be a part of the interrelated stress response system to manage the membrane. An increased permeability of IM caused by the mislocalization of secretin proteins activates the psp-system. Secretins are components of the export system essential for virulence, biofilm formation, and antibiotic resistance of EHEC (Joly et al., 2010; Srivastava et al., 2017). Under non-stress conditions, Psp proteins (except PspA) are almost undetectable where PspA along with HtpX and DnaK represses the psp-system (Elderkin et al., 2002). When a stress signal is received by IM-bound PspB and PspC and transferred to PspA thereby downregulating it and increasing other Psp proteins (Weiner et al., 1991; Jovanovic et al., 2006; Nonaka et al., 2006). In this study, we observed an upregulation in all *psp* (*pspA*, *pspB*, and *pspC*) genes, while PspA was expected to be downregulated in CFCSs-induced stress conditions. However, researchers also found that when any gene is only expressed under adaptive control (in this study *pspB* and *pspC*), there might be a delay to produce enough protein to adjust to the situation and during such time could lead to death or abnormal cellular morphology (Brissette et al., 1990; Price et al., 2013).

To evaluate the effect of CFCSs on the EHEC EDL933 cell membrane, we studied the EHEC membrane intactness and found evidence of membrane disruption caused by metabolites present in CFCSs. Our fluorescent microscopic and spectrophotometric data indicated that during the earlier hours of treatment, membrane disruption was less which was apparent by the lower signal intensity of red-fluorophore, PI. The PI is a widely used stain to detect dead cells; this dye can only penetrate cells with a disrupted membrane, intercalates to DNA irrespective of base-composition, or degradation with one dye molecule binding per 4–5 base pairs (Waring, 1965; Stocks, 2004). In our study, the proportion of membrane damaged cells increased with longer CFCS treatment duration, albeit there was a considerable proportion of green-fluorescent signal from SYTO 9 dye indicating the presence of bacteria with intact membrane. This variance of our fluorescent microscopic observation from the time-dependent growth reduction of EHEC was understood after the EHEC membrane morphology analysis by SEM. The electron micrographs revealed several membrane altering effects of CFCSs on EHEC. The control cells displayed a normal intact surface whereas the treatments exhibited a multitude of the wrinkled-irregular membrane, bleb-like surface, deep craters on both sides of the cell, shrinkage of the IM, blisters, etc. The “bleby” surface morphology was observed uniquely in CFCS^wt+PF^ treatment. This type of structure is formed at places on the outer membrane due to the deficiency of lysine, which can result in the detachment of the outer membrane from the underlying layer of peptidoglycan (Chatterjee and Chaudhuri, 2012). Lysine is an essential amino acid for humans which is present in peanuts and is also required for bacterial membrane protein synthesis (Gillner et al., 2013). Peanut originated components later metabolized by *L. casei* in our study have presumptively interfered with the EHEC lysine biosynthetic pathway hence resulted in the bleb-like appearance. In all treatment, no obvious disruption was apparent in some cells other than a detachment of the IM from the outer membrane, which explains the dissimilarity of our fluorescent microscopic and EHEC EDL933 growth reduction observation.

Heat shock proteins induced under stress usually act as protease (e.g., HtpX, Lon, and DegP), chaperons (e.g., ClpB, DnaJ, and DnaK), or chaperon helper (e.g., IbpA and IbpB) (Lewis, 2019). We observed the expression of some SOS pathway and chaperon related genes of EHEC EDL933 in the presence of collected CFCSs. LexA and RecA are two major proteins of SOS response in EHEC EDL933 where the cell’s response to DNA damage is mediated by expression of about 40 genes of SOS regulon by LexA repressor and RecA (Campoy et al., 2005). In normal growth condition, LexA represses/limit the expression of SOS genes (Friedberg et al., 2005). Upon encountering DNA damage stimuli, *RecA* becomes activated which facilitates the autolysis of LexA thereby initiating the expression of SOS genes that are involved in DNA damage tolerance, DNA repair, and delay of the cell cycle (Kreuzer, 2013). In our study, we observed a parallel overexpression of both *lexA* and *recA*. Upregulation of another two genes *tus* and *yebG* which are related to stopping DNA replication by blocking the movement of the replication fork and assisting in SOS response, respectively, indicated a DNA damaging stimuli presented by the CFCSs components (Roecklein et al., 1991; Lomba et al., 1997). Following the gene expression results of this study, we observed the DNA damaging effect of CFCS on EHEC EDL933 genomic DNA on the agarose gel. Such an effect could be attributed to the acidic pH of the CFCSs, but the same outcome was observed with pH adjusted CFCSs. So, the DNA damaging effect is linked to the specific compounds of CFCS possibly H_2_O_2_ (Brudzynski et al., 2012). The residual product size of the cleaved DNA on agarose gel poses an interesting observation that requires further investigation to reveal their sequences.

Molecular chaperones are proteins that assist a wide range of other proteins and enzymes to ensure their correct tertiary and quaternary conformation and functionality *in vivo* (Bukau et al., 2000; Saibil, 2013). All living cells constitutively maintain an intense regulation of chaperon gene expression to maintain consistency in protein folding to cope up with any growth perturbation (Morimoto, 1998). Therefore, chaperones or heat shock proteins are always expressed but expressed differently during stress conditions. In this study, an upregulation of the chaperon gene expression (*ibpA*, *ibpB*, *clpB*, *grpE*, *dnaJ*, and *dnaK*) in the presence of CFCSs was observed. Our findings in chaperon gene expression indicated stressed conditions created by CFCSs. However, as chaperones are involved in multiple complex cross-talks that are active in various conditions, further extensive analysis is needed to evaluate whether the upregulation is directed by solely CFCS-induced stress conditions.

Though evaluating the mechanism of total CFCS rather than finding out the individual metabolite responsible for exerting the antibacterial effect on EHEC is a limitation, but to our knowledge, this is the first study to address the mechanism of action of metabolites produced by probiotics against foodborne *E. coli* O157: H7. Further in-depth and extensive research is needed to find out the responsible metabolites by investigating specific protein-ligand interaction through combining different computational approaches. The orchestrated effect of metabolites present in the CFCSs of probiotics on the membrane and genomic DNA of EHEC could be potentially targeted to develop a therapeutic for EHEC infections including HUS.

## Data Availability Statement

The raw data supporting the conclusions of this article will be made available by the authors, without undue reservation.

## Author Contributions

AA designed and performed experiments, interpreted and analyzed data, ensured the integrity of the work, and prepared and revised the manuscript. MP ensured the accuracy of the work and revised the manuscript. AY performed experiments and proofread the manuscript. DB contributed to the conception and plan of the project and ensured both the accuracy and integrity of the work and critically revised and approved the final manuscript for submission and publication. All authors contributed to the article and approved the submitted version.

## Conflict of Interest

The authors declare that the research was conducted in the absence of any commercial or financial relationships that could be construed as a potential conflict of interest.

## References

[B1] Achar P. N. Hermetz K. Rao S. Apkarian R. Taylor J. (2009). Microscopic studies on the *Aspergillus flavus* infected kernels of commercial peanuts in Georgia. *Ecotoxicol. Environ. Saf.* 72 2115–2120. 10.1016/j.ecoenv.2009.04.002 19443032

[B2] Addinall S. G. Cao C. Lutkenhaus J. (1997). Temperature shift experiments with an ftsZ84(Ts) strain reveal rapid dynamics of *FtsZ* localization and indicate that the Z ring is required throughout septation and cannot reoccupy division sites once constriction has initiated. *J. Bacteriol.* 179 4277–4284. 10.1128/jb.179.13.4277-4284.1997 9209044 PMC179250

[B3] Aditya A. Alvarado-Martinez Z. Nagarajan V. Peng M. Biswas D. (2019). Antagonistic effects of phenolic extracts of Chokeberry pomace on *E. coli* O157: H7 but not on probiotic and normal bacterial flora. *J. Berry Res.* 9 459–472. 10.3233/jbr-190383

[B4] Arends S. J. R. Weiss D. S. (2004). Inhibiting cell division in *Escherichia coli* has little if any effect on gene expression. *J. Bacteriol.* 186 880–884. 10.1128/jb.186.3.880-884.2004 14729718 PMC321490

[B5] Arya S. S. Salve A. R. Chauhan S. (2016). Peanuts as functional food: a review. *J. Food Sci. Technol.* 53 31–41. 10.1007/s13197-015-2007-9 26787930 PMC4711439

[B6] Banni S. (2002). Conjugated linoleic acid metabolism. *Curr. Opin. Lipidol.* 13 261–266. 12045395 10.1097/00041433-200206000-00005

[B7] Barnett D. Baldo B. A. Howden M. E. H. (1983). Multiplicity of allergens in peanuts. *J. Allergy Clin. Immunol.* 72 61–68. 10.1016/0091-6749(83)90053-2 6853931

[B8] Bell B. P. Griffin P. M. Lozano P. Christie D. L. Kobayashi J. M. Tarr P. I. (1997). Predictors of hemolytic uremic syndrome in children during a large outbreak of *Escherichia coli* O157:H7 infections. *Pediatrics* 100:E12. 10.1542/peds.100.1.e12 9200386

[B9] Belury M. A. (2002). Inhibition of carcinogenesis by conjugated linoleic acid: potential mechanisms of action. *J. Nutr.* 132 2995–2998. 10.1093/jn/131.10.2995 12368384

[B10] Bernet M. F. Brassart D. Neeser J. R. Servin A. L. (1994). *Lactobacillus acidophilus* LA 1 binds to cultured human intestinal cell lines and inhibits cell attachment and cell invasion by enterovirulent bacteria. *Gut* 35 483–489. 10.1136/gut.35.4.483 8174985 PMC1374796

[B11] Brissette J. L. Russel M. Weiner L. Model P. (1990). Phage shock protein, a stress protein of *Escherichia coli*. *Proc. Natl. Acad. Sci. U.S.A.* 87 862–866. 2105503 10.1073/pnas.87.3.862 PMC53368

[B12] Broberg A. Jacobsson K. Ström K. Schnürer J. (2007). Metabolite profiles of lactic acid bacteria in grass silage. *Appl. Environ. Microbiol.* 73 5547–5552. 10.1128/aem.02939-06 17616609 PMC2042065

[B13] Brudzynski K. Abubaker K. Miotto D. (2012). Unraveling a mechanism of honey antibacterial action: polyphenol/H2O2-induced oxidative effect on bacterial cell growth and on DNA degradation. *Food Chem.* 133 329–336. 10.1016/j.foodchem.2012.01.035 25683403

[B14] Bukau B. Deuerling E. Pfund C. Craig E. A. (2000). Getting newly synthesized proteins into shape. *Cell* 101 119–122. 10.1016/s0092-8674(00)80806-5 10786831

[B15] Callaway T. R. Anderson R. C. Edrington T. S. Genovese K. J. Harvey R. B. Poole T. L. (2013). *Advances in Microbial Food Safety: 14. Novel Methods for Pathogen Control in Livestock Pre-Harvest: an Update.* Amsterdam: Elsevier.

[B16] Campoy S. Salvador N. Cortés P. Erill I. Barbé J. (2005). Expression of canonical SOS genes is not under LexA repression in *Bdellovibrio bacteriovorus*. *J. Bacteriol.* 187 5367–5375. 10.1128/jb.187.15.5367-5375.2005 16030231 PMC1196036

[B17] Cardenal-Muñoz E. Ramos-Morales F. (2011). Analysis of the expression, secretion and translocation of the *Salmonella enterica* type III secretion system effector SteA. *PLoS One* 6:e26930. 10.1371/journal.pone.0026930 22046414 PMC3203157

[B18] Charernjiratragul W. Bhoopong P. Kantachote D. Jomduang S. Kong-Ngoen R. Nair G. B. (2010). Inhibitory activity of lactic acid bacteria isolated from Thai fermented food against pandemic strains of *Vibrio parahaemolyticus*. *J. Food Saf.* 30 67–82. 10.1111/j.1745-4565.2009.00190.x

[B19] Chatterjee S. N. Chaudhuri K. (2012). *Outer Membrane Vesicles of Bacteria.* Berlin: Springer Science+Business Media.

[B20] Chomczynski P. Sacchi N. (1987). Single-step method of RNA isolation by acid guanidinium thiocyanate-phenol-chloroform extraction. *Anal. Biochem.* 162 156–159. 10.1006/abio.1987.9999 2440339

[B21] Cimolai N. Morrison B. J. Carter J. E. (1992). Risk factors for the central nervous system manifestations of gastroenteritis-associated hemolytic-uremic syndrome. *Pediatrics* 90 616–621. 1408519

[B22] Cleveland J. Montville T. J. Nes I. F. Chikindas M. L. (2001). Bacteriocins: safe, natural antimicrobials for food preservation. *Int. J. Food Microbiol.* 71 1–20. 10.1016/s0168-1605(01)00560-8 11764886

[B23] Cordonnier C. Etienne-Mesmin L. Thévenot J. Rougeron A. Rénier S. Chassaing B. (2017). Enterohemorrhagic *Escherichia coli* pathogenesis: role of long polar fimbriae in Peyer’s patches interactions. *Sci. Rep.* 7:44655. 10.1038/srep44655 PMC5357955 28317910

[B24] Delgado A. Arroyo López F. N. Brito D. Peres C. Fevereiro P. Garrido-Fernández A. (2007). Optimum bacteriocin production by *Lactobacillus plantarum* 17.2b requires absence of NaCl and apparently follows a mixed metabolite kinetics. *J. Biotechnol.* 130 193–201. 10.1016/j.jbiotec.2007.01.041 17462774

[B25] Drexler M. Institute of Medicine (US) (2010). *Prevention and Treatment.* Washington, DC: National Academies Press.

[B26] Elderkin S. Jones S. Schumacher J. Studholme D. Buck M. (2002). Mechanism of action of the *Escherichia coli* phage shock protein PspA in repression of the AAA family transcription factor PspF. *J. Mol. Biol.* 320 23–37. 10.1016/s0022-2836(02)00404-7 12079332

[B27] Farkas A. Maróti G. Kereszt A. Kondorosi É. (2017). Comparative analysis of the bacterial membrane disruption effect of two natural plant antimicrobial peptides. *Front. Microbiol.* 8:51. 10.3389/fmicb.2017.00051 28167938 PMC5253368

[B28] Friedberg E. C. Walker G. C. Siede W. Wood R. D. (2005). *DNA Repair and Mutagenesis.* Washington, DC: American Society for Microbiology Press.

[B29] Fuller R. (1989). Probiotics in man and animals. *J. Appl. Bacteriol.* 66 365–378. 10.1111/j.1365-2672.1989.tb05105.x 2666378

[B30] Gillner D. M. Becker D. P. Holz R. C. (2013). Lysine biosynthesis in bacteria: a metallodesuccinylase as a potential antimicrobial target. *J. Biol. Inorg. Chem.* 18 155–163. 10.1007/s00775-012-0965-1 23223968 PMC3862034

[B31] Grosso G. Yang J. Marventano S. Micek A. Galvano F. Kales S. N. (2015). Nut consumption on all-cause, cardiovascular, and cancer mortality risk: a systematic review and meta-analysis of epidemiologic studies. *Am. J. Clin. Nutr.* 101 783–793. 10.3945/ajcn.114.099515 25833976

[B32] Johns C. O. Jones D. B. (1916). The proteins of the peanut, Arachis hypogæa I. the globulins arachin and conarachin. *J. Biol. Chem.* 28 77–87.

[B33] Joly N. Engl C. Jovanovic G. Huvet M. Toni T. Sheng X. (2010). Managing membrane stress: the phage shock protein (Psp) response, from molecular mechanisms to physiology. *FEMS Microbiol. Rev.* 34 797–827. 10.1111/j.1574-6976.2010.00240.x 20636484

[B34] Jonas K. (2014). To divide or not to divide: control of the bacterial cell cycle by environmental cues. *Curr. Opin. Microbiol.* 18 54–60. 10.1016/j.mib.2014.02.006 24631929

[B35] Jones R. J. Hussein H. M. Zagorec M. Brightwell G. Tagg J. R. (2008). Isolation of lactic acid bacteria with inhibitory activity against pathogens and spoilage organisms associated with fresh meat. *Food Microbiol.* 25 228–234. 10.1016/j.fm.2007.11.001 18206764

[B36] Jovanovic G. Lloyd L. J. Stumpf M. P. H. Mayhew A. J. Buck M. (2006). Induction and function of the phage shock protein extracytoplasmic stress response in *Escherichia coli*. *J. Biol. Chem.* 281 21147–21161. 10.1074/jbc.m602323200 16709570

[B37] Kechagia M. Basoulis D. Konstantopoulou S. Dimitriadi D. Gyftopoulou K. Skarmoutsou N. (2013). Health benefits of probiotics: a review. *ISRN Nutr.* 2013:481651. 10.5402/2013/481651 PMC4045285 24959545

[B38] Kishino S. Ogawa J. Omura Y. Matsumura K. Shimizu S. (2002). Conjugated linoleic acid production from linoleic acid by lactic acid bacteria. *J. Am. Oil Chem. Soc.* 79 159–163. 10.1007/s11746-002-0451-4

[B39] Kreuzer K. N. (2013). DNA damage responses in prokaryotes: regulating gene expression, modulating growth patterns, and manipulating replication forks. *Cold Spring Harb. Perspect. Biol.* 5:a012674. 10.1101/cshperspect.a012674 24097899 PMC3809575

[B40] Lamuel-Raventos R. M. Onge M. P. S. (2017). Prebiotic nut compounds and human microbiota. *Crit. Rev. Food Sci. Nutr.* 57 3154–3163. 10.1080/10408398.2015.1096763 27224877 PMC5646185

[B41] Lewis K. (2019). *Persister Cells and Infectious Disease.* London: Springer Nature.

[B42] Livak K. J. Schmittgen T. D. (2001). Analysis of relative gene expression data using real-time quantitative PCR and the 2(-Delta Delta C(T)) Method. *Methods* 25 402–408. 10.1006/meth.2001.1262 11846609

[B43] Llor C. Bjerrum L. (2014). Antimicrobial resistance: risk associated with antibiotic overuse and initiatives to reduce the problem. *Ther. Adv. Drug Saf.* 5 229–241. 10.1177/2042098614554919 25436105 PMC4232501

[B44] Lomba M. R. Vasconcelos A. T. Pacheco A. B. de Almeida D. F. (1997). Identification of yebG as a DNA damage-inducible *Escherichia coli* gene. *FEMS Microbiol. Lett.* 156 119–122. 10.1111/j.1574-6968.1997.tb12715.x 9368369

[B45] Matsuo Y. Miyoshi Y. Okada S. Satoh E. (2012). Receptor-like molecules on human intestinal epithelial cells interact with an adhesion factor from *Lactobacillus reuteri*. *Biosci. Microbiota Food Health* 31 93–102. 10.12938/bmfh.31.93 24936355 PMC4034283

[B46] Mercenier A. Lenoir-Wrjnkoop I. Sanders M. E. (2008). Physiological and functional properties of probiotics. *J. Milk Sci. Biotechnol.* 26 53–57.

[B47] Mezaini A. Chihib N.-E. Dilmi Bouras A. Nedjar-Arroume N. Hornez J. P. (2009). Antibacterial activity of some lactic acid bacteria isolated from an algerian dairy product. *J. Environ. Public Health* 2009:678495. 10.1155/2009/678495 PMC2778462 20041021

[B48] Ming T. Han J. Li Y. Lu C. Qiu D. Li Y. (2018). A metabolomics and proteomics study of the *Lactobacillus plantarum* in the grass carp fermentation. *BMC Microbiol.* 18:216. 10.1186/s12866-018-1354-x 30563460 PMC6299570

[B49] Morimoto R. I. (1998). Regulation of the heat shock transcriptional response: cross talk between a family of heat shock factors, molecular chaperones, and negative regulators. *Genes Dev.* 12 3788–3796. 10.1101/gad.12.24.3788 9869631

[B50] Murray M. D. Brater D. C. (1993). Renal toxicity of the nonsteroidal anti-inflammatory drugs. *Annu. Rev. Pharmacol. Toxicol.* 33 435–465. 8494347 10.1146/annurev.pa.33.040193.002251

[B51] Nigatu J. M. Tuji F. A. Tefera A. T. (2015). Evaluation of the antagonistic effect of six mixed cultures of lactic acid bacteria, isolated from the Ethiopian fermented milk ergo, against some foodborne pathogens inoculated into the Ethiopian cottage cheese ayib. *Afr. J. Microbiol. Res.* 9 1789–1797. 10.5897/ajmr2015.7504

[B52] Nonaka G. Blankschien M. Herman C. Gross C. A. Rhodius V. A. (2006). Regulon and promoter analysis of the *E. coli* heat-shock factor, σ32, reveals a multifaceted cellular response to heat stress. *Genes Dev.* 20 1776–1789. 10.1101/gad.1428206 16818608 PMC1522074

[B53] Ogawa J. Matsumura K. Kishino S. Omura Y. Shimizu S. (2001). Conjugated linoleic acid accumulation via 10-hydroxy-12-octadecaenoic acid during microaerobic transformation of linoleic acid by *Lactobacillus acidophilus*. *Appl. Environ. Microbiol.* 67 1246–1252. 10.1128/aem.67.3.1246-1252.2001 11229917 PMC92720

[B54] Ouwehand A. C. Salminen S. Isolauri E. (2002). Probiotics: an overview of beneficial effects. *Antonie Van Leeuwenhoek* 82 279–289. 10.1007/978-94-017-2029-8_18 12369194

[B55] Peng M. Biswas D. (2017). Short chain and polyunsaturated fatty acids in host gut health and foodborne bacterial pathogen inhibition. *Crit. Rev. Food Sci. Nutr.* 57 3987–4002. 10.1080/10408398.2016.1203286 27438132

[B56] Peng M. Bitsko E. Biswas D. (2015a). Functional properties of peanut fractions on the growth of probiotics and foodborne bacterial pathogens. *J. Food Sci.* 80 M635–M641. 25627431 10.1111/1750-3841.12785

[B57] Peng M. Reichmann G. Biswas D. (2015b). *Lactobacillus casei* and its byproducts alter the virulence factors of foodborne bacterial pathogens. *J. Funct. Foods* 15 418–428. 10.1016/j.jff.2015.03.055

[B58] Peng M. Tabashsum Z. Patel P. Bernhardt C. Biswas D. (2018). Linoleic acids overproducing *Lactobacillus casei* limits growth, survival, and virulence of *Salmonella* typhimurium and enterohaemorrhagic *Escherichia coli*. *Front. Microbiol.* 9:2663. 10.3389/fmicb.2018.02663 30443248 PMC6223203

[B59] Pranckutė R. Kaunietis A. Kuisienė N. Čitavičius D. J. (2016). Combining prebiotics with probiotic bacteria can enhance bacterial growth and secretion of bacteriocins. *Int. J. Biol. Macromol.* 89 669–676. 10.1016/j.ijbiomac.2016.05.041 27181578

[B60] Price M. N. Deutschbauer A. M. Skerker J. M. Wetmore K. M. Ruths T. Mar J. S. (2013). Indirect and suboptimal control of gene expression is widespread in bacteria. *Mol. Syst. Biol.* 9:660. 10.1038/msb.2013.16 23591776 PMC3658271

[B61] Ramalingum N. Mahomoodally M. F. (2014). The therapeutic potential of medicinal foods. *Adv. Pharmacol. Sci.* 2014:354264. 10.1155/2014/354264 PMC4009199 24822061

[B62] Reid G. McCormick J. K. (2002). Time for probiotics. *Lancet Infect. Dis.* 2:459. 10.1016/s1473-3099(02)00340-7 12150842

[B63] Ren D. Zhu J. Gong S. Liu H. Yu H. (2018). Antimicrobial characteristics of lactic acid bacteria isolated from homemade fermented foods. *Biomed Res. Int.* 2018:5416725. 10.1155/2018/5416725 PMC6330816 30687749

[B64] Roecklein B. Pelletier A. Kuempel P. (1991). The *tus* gene of *Escherichia coli*: autoregulation, analysis of flanking sequences and identification of a complementary system in *Salmonella* typhimurium. *Res. Microbiol.* 142 169–175. 10.1016/0923-2508(91)90026-7 1925016

[B65] Rurangwa E. Laranja J. L. Houdt R. V. Delaedt Y. Geraylou Z. de Wiele T. V. (2009). Selected nondigestible carbohydrates and prebiotics support the growth of probiotic fish bacteria mono-cultures in vitro. *J. Appl. Microbiol.* 106 932–940. 10.1111/j.1365-2672.2008.04034.x 19191975

[B66] Saarela M. Mogensen G. Fondén R. Mättö J. Mattila-Sandholm T. (2000). Probiotic bacteria: safety, functional and technological properties. *J. Biotechnol.* 84 197–215. 10.1016/s0168-1656(00)00375-8 11164262

[B67] Saibil H. (2013). Chaperone machines for protein folding, unfolding and disaggregation. *Nat. Rev. Mol. Cell Biol.* 14 630–642. 10.1038/nrm3658 24026055 PMC4340576

[B68] Sakoh M. Ito K. Akiyama Y. (2005). Proteolytic activity of HtpX, a membrane-bound and stress-controlled protease from *Escherichia coli*. *J. Biol. Chem.* 280 33305–33310. 10.1074/jbc.m506180200 16076848

[B69] Salaheen S. Jaiswal E. Joo J. Peng M. Ho R. OConnor D. (2016). Bioactive extracts from berry byproducts on the pathogenicity of *Salmonella* typhimurium. *Int. J. Food Microbiol.* 237 128–135. 10.1016/j.ijfoodmicro.2016.08.027 27565525

[B70] Salminen S. J. Gueimonde M. Isolauri E. (2005). Probiotics that modify disease risk. *J. Nutr.* 135 1294–1298. 10.1093/jn/135.5.1294 15867327

[B71] Schnürer J. Magnusson J. (2005). Antifungal lactic acid bacteria as biopreservatives. *Trends Food Sci. Technol.* 16 70–78. 10.1016/j.tifs.2004.02.014

[B72] Schrezenmeir J. de Vrese M. (2001). Probiotics, prebiotics, and synbiotics—approaching a definition. *Am. J. Clin. Nutr.* 73 361s–364s. 10.1093/ajcn/73.2.361s 11157342

[B73] Seshasayee A. S. N. Fraser G. M. Babu M. M. Luscombe N. M. (2009). Principles of transcriptional regulation and evolution of the metabolic system in *E. coli*. *Genome Res.* 19 79–91. 10.1101/gr.079715.108 18836036 PMC2612968

[B74] Shimohata N. Chiba S. Saikawa N. Ito K. Akiyama Y. (2002). The Cpx stress response system of *Escherichia coli* senses plasma membrane proteins and controls HtpX, a membrane protease with a cytosolic active site. *Genes Cells* 7 653–662. 10.1046/j.1365-2443.2002.00554.x 12081643

[B75] Sorroche F. G. Spesia M. B. Zorreguieta Á. Giordano W. (2012). A positive correlation between bacterial autoaggregation and biofilm formation in native *Sinorhizobium meliloti* isolates from Argentina. *Appl. Environ. Microbiol.* 78 4092–4101. 10.1128/aem.07826-11 22492433 PMC3370541

[B76] Srivastava D. Moumene A. Flores-Kim J. Darwin A. J. (2017). Psp stress response proteins form a complex with mislocalized secretins in the *Yersinia enterocolitica* cytoplasmic membrane. *mBio* 8:e01088-17. 10.1128/mBio.01088-17 PMC5596341 28900025

[B77] Stocks S. M. (2004). Mechanism and use of the commercially available viability stain, BacLight. *Cytometry A* 61 189–195. 10.1002/cyto.a.20069 15382024

[B78] Tabashsum Z. Peng M. Salaheen S. Comis C. Biswas D. (2018). Competitive elimination and virulence property alteration of *Campylobacter jejuni* by genetically engineered *Lactobacillus casei*. *Food Control* 85 283–291. 10.1016/j.foodcont.2017.10.010

[B79] Ventola C. L. (2015). The antibiotic resistance crisis. *Pharm. Ther.* 40 277–283. PMC4378521 25859123

[B80] Vicente M. Rico A. I. Martínez-Arteaga R. Mingorance J. (2006). Septum enlightenment: assembly of bacterial division proteins. *J. Bacteriol.* 188 19–27. 10.1128/jb.188.1.19-27.2006 16352817 PMC1317574

[B81] Vu B. Chen M. Crawford R. J. Ivanova E. P. (2009). Bacterial extracellular polysaccharides involved in biofilm formation. *Molecules* 14 2535–2554. 10.3390/molecules14072535 19633622 PMC6254922

[B82] Wall M. E. Hlavacek W. S. Savageau M. A. (2004). Design of gene circuits: lessons from bacteria. *Nat. Rev. Genet.* 5 34–42. 10.1038/nrg1244 14708014

[B83] Waring M. J. (1965). Complex formation between ethidium bromide and nucleic acids. *J. Mol. Biol.* 13 269–282. 10.1016/s0022-2836(65)80096-1 5859041

[B84] Weiner L. Brissette J. L. Model P. (1991). Stress-induced expression of the *Escherichia coli* phage shock protein operon is dependent on sigma 54 and modulated by positive and negative feedback mechanisms. *Genes Dev.* 5 1912–1923. 10.1101/gad.5.10.1912 1717346

[B85] Yoshitani K. Hizukuri Y. Akiyama Y. (2019). An in vivo protease activity assay for investigating the functions of the *Escherichia coli* membrane protease HtpX. *FEBS Lett.* 593 842–851. 10.1002/1873-3468.13368 30903618

